# Quantitative super-resolution microscopy reveals the differences in the nanoscale distribution of nuclear phosphatidylinositol 4,5-bisphosphate in human healthy skin and skin warts

**DOI:** 10.3389/fcell.2023.1217637

**Published:** 2023-07-07

**Authors:** Peter Hoboth, Martin Sztacho, Alexander Quaas, Baki Akgül, Pavel Hozák

**Affiliations:** ^1^ Department of Biology of the Cell Nucleus, Institute of Molecular Genetics of the Czech Academy of Sciences, Prague, Czechia; ^2^ Institute of Pathology, Medical Faculty and University Hospital Cologne, Cologne, Germany; ^3^ Institute of Virology, University of Cologne, Medical Faculty and University Hospital Cologne, Cologne, Germany; ^4^ Microscopy Centre, Institute of Molecular Genetics of the Czech Academy of Sciences, Prague, Czechia

**Keywords:** formalin-fixed paraffin-embedded tissue sections, STED nanoscopy, cell nucleus, human papillomavirus (HPV), nuclear architecture, phosphatidylinositol 4,5-bisphosphate, nuclear speckles, quantitative image analysis

## Abstract

**Introduction:** Imaging of human clinical formalin-fixed paraffin-embedded (FFPE) tissue sections provides insights into healthy and diseased states and therefore represents a valuable resource for basic research, as well as for diagnostic and clinical purposes. However, conventional light microscopy does not allow to observe the molecular details of tissue and cell architecture due to the diffraction limit of light. Super-resolution microscopy overcomes this limitation and provides access to the nanoscale details of tissue and cell organization.

**Methods:** Here, we used quantitative multicolor stimulated emission depletion (STED) nanoscopy to study the nanoscale distribution of the nuclear phosphatidylinositol 4,5-bisphosphate (nPI(4,5)P2) with respect to the nuclear speckles (NS) marker SON.

**Results:** Increased nPI(4,5)P2 signals were previously linked to human papillomavirus (HPV)-mediated carcinogenesis, while NS-associated PI(4,5)P2 represents the largest pool of nPI(4,5)P2 visualized by staining and microscopy. The implementation of multicolor STED nanoscopy in human clinical FFPE skin and wart sections allowed us to provide here the quantitative evidence for higher levels of NS-associated PI(4,5)P2 in HPV-induced warts compared to control skin.

**Discussion:** These data expand the previous reports of HPV-induced increase of nPI(4,5)P2 levels and reveal for the first time the functional, tissue-specific localization of nPI(4,5)P2 within NS in clinically relevant samples. Moreover, our approach is widely applicable to other human clinical FFPE tissues as an informative addition to the classical histochemistry.

## 1 Introduction

Human tissues have been collected and stored in biobanks for more than 100 years for educational and research purposes. At the turn of the 20th century more than 300 million of tissue specimens were stored only in the United States and accumulating at a rate of more than 20 million per year (Baker, 2012; Eiseman and Haga, 1999). The most practical way of archiving clinical samples is formalin fixation and paraffin embedding (FFPE), which preserves tissues for extended periods even at ambient temperatures (Ilgen et al., 2014; Lou et al., 2014). Visualization of morphological features by conventional light microscopy is still the most frequently used method for disease diagnosis and analysis of pathological hallmarks. For this purpose, FFPE tissue samples are first sectioned, mostly into the 4 µm thick sections, dewaxed and then stained. Immunohistochemistry, immunofluorescence, molecular profiling using *in situ* hybridization and other techniques are commonly performed using FFPE samples (Kokkat et al., 2013). Nevertheless, the diffraction limit of light curtails the detailed investigation of biological specimens by optical microscopy as it allows distinguishing objects only if they are ~ 200 nm apart (Abbe, 1873; Rayleigh, 1896). The resolution limit depends on the wavelength and therefore it is possible to improve it using electrons instead of light (Rayleigh, 1896). Hence, if ultrastructural resolution is desired, pathology traditionally employs electron microscopy (Peddie and Collinson, 2014; Pinali and Kitmitto, 2014). However, it has significant limitations in routine clinical use, including cost, slow sample preparation, and limitations in multi-component and 3D imaging.

The invention of super-resolution microscopy (SRM) allowed to overcome the diffraction limit in optical microscopy and thereby enabled the resolution of fluorescently labeled molecules at the nanoscale (Schermelleh et al., 2010; Liu et al., 2015; Tam and Merino, 2015; Turkowyd et al., 2016). SRM achieves sub-diffraction limited resolution by either stochastic or deterministic temporal control of the fluorescence emission from only a subset of fluorophores from the total fluorophore population in the specimen. Stochastic control of fluorophore emission is the basis for single-molecule localization microscopy (SMLM), such as direct stochastic optical reconstruction microscopy (dSTORM) (Heilemann et al., 2008; van de Linde et al., 2011). Previously, single-color dSTORM in FFPE human breast cancer tissue provided insight into the nanoscale organization of the cell membrane marker HER2, the outer mitochondrial membrane protein TOM20 and Lamin B1, a component of the nuclear envelope (Creech et al., 2017). Deterministic control of fluorophore emission is utilized in stimulated emission depletion (STED) microscopy (Hell and Wichmann, 1994; Klar et al., 2000). Single-color STED microscopy has been previously used to visualize the details of the surface and intracellular HER2 cancer marker distribution (Ilgen et al., 2014). These pioneering SRM studies of human FFPE tissue sections (Ilgen et al., 2014; Creech et al., 2017) were, however, limited to a single color and lacked comparisons between cancer and healthy tissues. Thus, their descriptive nature rather served as a proof of principle for SRM in human clinical FFPE tissue sections. Here, we extended these previous efforts and optimized multicolor STED nanoscopy in human FFPE clinical tissue sections and implemented subsequent quantitative analyses of the nanoscale functional organization of nuclear antigens.

Nuclear speckles (NS) are sub-nuclear compartments that were earlier called interchromatin granule clusters (Thiry, 1993). NS are mainly composed of the proteins SON and SRRM2, pre-mRNA splicing factors (SFs), small nuclear ribonucleoprotein particles (snRNPs) and poly(A)^+^ RNAs (Thiry, 1993; Mintz et al., 1999; Lamond and Spector, 2003; Saitoh et al., 2004; Hall et al., 2006; Spector and Lamond, 2011; Ilik et al., 2020; Ilik and Aktas, 2021). NS are involved in gene expression, including pre-mRNA processing and mRNA export (Prasanth et al., 2003; Brown et al., 2008; Berchtold et al., 2018; Chen et al., 2018; Chen and Belmont, 2019; Kim et al., 2020; Alexander et al., 2021). When visualized by microscopy, the largest pool of nuclear PI(4,5)P2 (nPI(4,5)P2) as well as the enzymes involved in its biosynthesis appear in NS (Boronenkov et al., 1998; Osborne et al., 2001; Bunce et al., 2006; Mellman et al., 2008; Mellman and Anderson, 2009; Sobol et al., 2018; Hoboth et al., 2021c), suggesting a role of NS in the nPI(4,5)P2 metabolism. An earlier quantitative dual-color SRM study revealed specific co-patterning between nPI(4,5)P2 and the NS marker SON (Hoboth et al., 2021c). PI(4,5)P2 is a powerful signaling molecule with a plethora of functions ranging from the cell membrane to the nucleus (Hammond et al., 2004; Fiume et al., 2012; Balla, 2013; Shah et al., 2013). Nuclear PI(4,5)P2 play roles in the nuclear compartmentalization (Mellman and Anderson, 2009; Fiume et al., 2012; Shah et al., 2013; Castano et al., 2019; Sztacho et al., 2019), in the gene expression (Cocco et al., 1987; Divecha et al., 1991; Mazzotti et al., 1995; Zhao et al., 1998; York et al., 1999; Bunce et al., 2006; Mellman et al., 2008; Lewis et al., 2011; Sobol et al., 2013; Yildirim et al., 2013; Sobol et al., 2018; Balaban et al., 2023) and its interactors include NS-associated proteins (Saitoh et al., 2004; Lewis et al., 2011; Jacobsen et al., 2019; Balaban et al., 2021; Sztacho et al., 2021). Nevertheless, the precise link between NS functions and nPI(4,5)P2 remains elusive. Moreover, the visualization of SON within the tissue was so far limited to only few reports (George-Tellez et al., 2002; Saitoh et al., 2012; Kuga et al., 2016) and the information about nPI(4,5)P2 within the context of tissue is missing.

Interestingly, we recently described increased levels of nPI(4,5)P2 in mucosal and cutaneous squamous cell carcinoma (SCC) associated with infections by oncogenic human papillomaviruses (HPV). We therefore speculate that the increased nPI(4,5)P2 levels are a hallmark of HPV-induced tumorigenesis (Marx et al., 2018). These findings raised important questions regarding the role of nPI(4,5)P2 in epidermal tumorigenesis, including whether HPV types without oncogenic potential can also impact the nPI(4,5)P2. Chronic HPV infection of the cutaneous skin can be asymptomatic or cause benign skin warts, premalignant actinic keratosis or malignant SCC (Howley and Pfister, 2015; Hufbauer and Akgül, 2017; McBride, 2022). Here, we quantitatively assessed the nanoscale spatial co-patterning between nPI(4,5)P2 and SON in normal human FFPE skin sections and compared it with their co-distribution in HPV-induced skin warts.

## 2 Results

### 2.1 Stimulated emission depletion microscopy allows for the multicolor super-resolved imaging of nuclear antigens in human clinical formalin-fixed paraffin-embedded skin tissue sections

**FIGURE 1:**
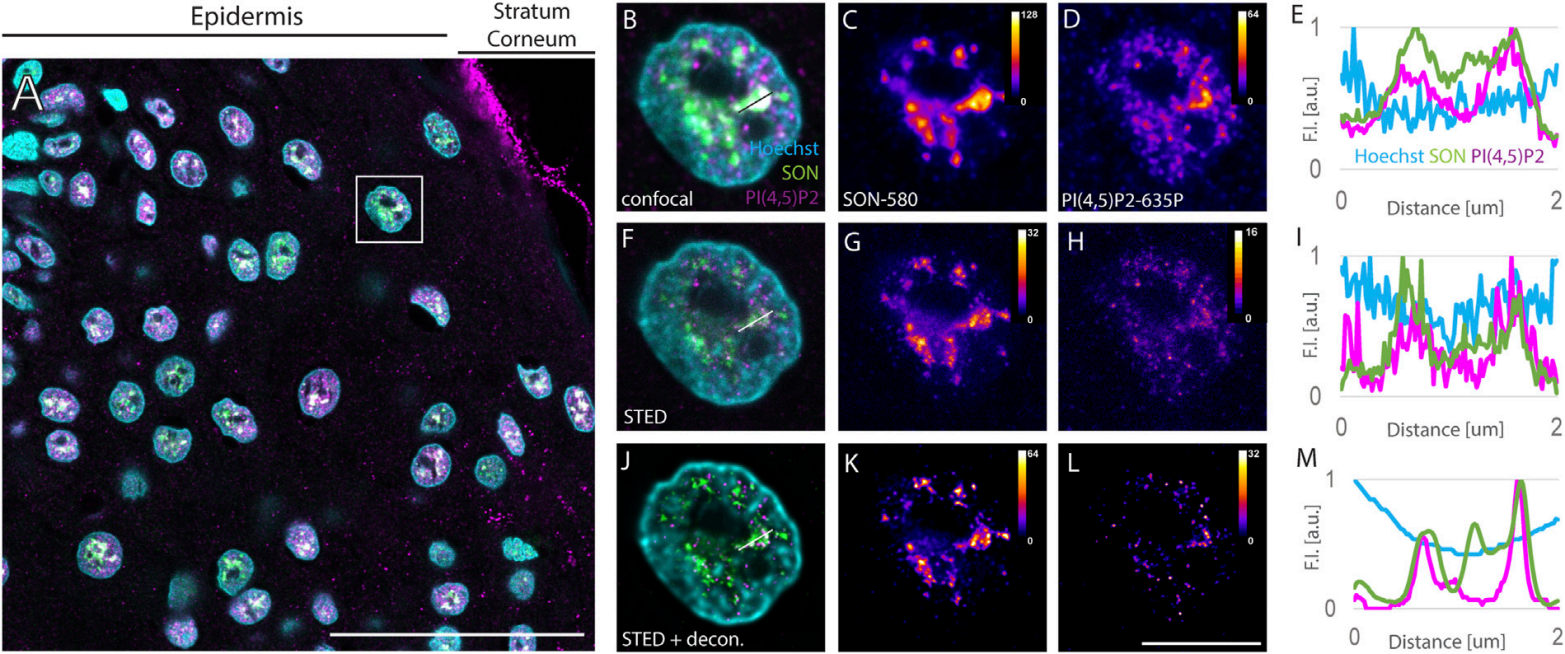
Imaging of nuclear antigens in human FFPE skin sections. Overview of the sample **(A)**, zoomed in to the boxed region **(B–L)**. Confocal **(B–D)** and STED **(F–H)** with deconvolution (J–L) images of the same nucleus with Hoechst nuclear stain in cyan, SON in green and PI(4,5)P2 in magenta **(A,B,F and J)**. Individual SON **(C,G and K)** and PI(4,5)P2 **(D, H, L)** channels in Fire LUT with corresponding calibration bars. FI line scan **(E, I, M)** along the line in (**B, F, J)**, resp. scale bar 50 µm **(A)** or 5 µm **(B–L)**.

Surgically removed human FFPE skin biopsies were sectioned into 4 µm sections. Sections were dewaxed, and indirectly immunofluorescently labeled against NS marker SON and nPI(4,5)P2. Primary antibodies against SON and PI(4,5)P2 were recognized by secondary antibodies conjugated with Abberior Star 580 and Abberior Star 635P, the fluorophores suitable for STED (Figure 1). Immunolabeled sections were then counterstained with Hoechst-JF503 to mark the nuclei. An overview of the epidermal part of the skin imaged by confocal microscopy showed Hoechst marked keratinocyte nuclei containing SON signals corresponding to NS and nPI(4,5)P2 (Figure 1A). Localization of nPI(4,5)P2 to NS was previously documented in cultured cells (Boronenkov et al., 1998; Mellman et al., 2008; Sobol et al., 2018; Hoboth et al., 2021b; Hoboth et al., 2021c; McBride, 2022), but not in the tissue. We visualized nPI(4,5)P2 using the previously validated antibody clone 2C11 (Boronenkov et al., 1998; Thomas et al., 1999; Mellman et al., 2008; Kalasova et al., 2016; Marx et al., 2018; Sobol et al., 2018; Balaban et al., 2021; Hoboth et al., 2021b; Hoboth et al., 2021c). In human FFPE skin sections, PI(4,5)P2 displays a predominantly nuclear signal mostly overlapping with the SON signal, but is also present in the nucleoplasm (Figures 1B–E). The stratum corneum (SC), which provides the barrier function of the skin (Menon et al., 2012), also shows PI(4,5)P2 signal (Figure 1A). This is consistent with the notion of SC being a lipid matrix containing phospholipids (van Smeden et al., 2014a; van Smeden et al., 2014b; Sjovall et al., 2022).

Keratinocyte nuclei showed signals of the nuclear speckles marker SON, forming cloud-like accumulations in the confocal images (Figures 1B,C) similarly to the cultured human osteosarcoma U2OS cell line (Supplementary Figure S1). SON localized in areas negative for the Hoechst signal (Figures 1B,E), which is in line with the localization of NS to the interchromatin space (Thiry, 1993) and with the situation observed in U2OS cells (Supplementary Figure S1). Nevertheless, the diffraction-limited confocal microscopy neither enables to precisely distinguish individual NS, nor to distinguish sub-diffraction limited foci of nPI(4,5)P2 (Sobol et al., 2018; Hoboth et al., 2021b; Hoboth et al., 2021c). Therefore, we investigated whether the same sample imaged using confocal microscopy is suitable for STED microscopy. For this purpose, we used 775 nm STED laser to deplete both, Abberior Star 580 and Abberior Star 635P, fluorophores at the periphery of the scanning focal spot of either 580 nm or 635 nm excitation laser. Thereby we achieved sub-diffraction limited resolution in the individual nuclei of the FFPE tissue section (Figures 1F–H) previously imaged by confocal microscopy (Figures 1B–D). Comparison between the confocal images of the NS marker SON (Figure 1C) and nPI(4,5)P2 (Figure 1D) and STED images of the same antigens (Figure 1 G and H, resp.) illustrated the super-resolved details in the STED images (Figures 1C,D) that were hidden in the confocal images (Figures 1G,H). Fluorescence intensity (FI) profile of the STED image (Figure 1I) showed narrower FI peaks compared to the peaks in the FI profile of the confocal image (Figure 1E). This documented the improved resolution of the subnuclear details within FFPE tissue sections imaged by STED microscopy as compared to the confocal microscopy and demonstrated the feasibility of the STED super-resolution imaging in the clinical FFPE human tissue samples. Although the FI was generally reduced by STED (compare Figures 1C,D with Figures 1G,H resp.), deconvolution further improved the resolution of STED images.

Deconvolution is a mathematical operation that reassigns out-of-focus light to its origin and thereby improves the sharpness and contrast of the original images (Wallace et al., 2001; Sibarita, 2005; Bolte and Cordelieres, 2006). Therefore, we subjected the Z-stacks of STED images of individual keratinocyte nuclei to deconvolution using Huygens software (see Materials and Methods). Figures 1F–H shows the same nuclei as in Figures 1B–D with SON-580 (Figure 1G) and PI(4,5)P2-635 (Figure 1H) channels imaged by STED microscopy and subsequent deconvolution. A comparison of confocal images (Figures 1C,D), raw STED images (Figures 1G,H) and deconvoluted STED images (Figures 1K,L) documented super-resolved details of the sub-diffraction limited organization of the keratinocyte NS that were hindered when imaged by confocal microscopy. Deconvolution of STED images smoothened the FI profiles (Figure 1M) and suppressed the noise displayed by confocal (Figures 1B–E) as well as raw STED images (Figures 1F–I). Normalized FI line scan (Figure 1M) further illustrated improved resolution achieved by STED microscopy (Figure 1I) and subsequent deconvolution (Figure 1M) in the FFPE tissue sections compared to confocal microscopy (Figure 1E).

Taken together, we demonstrated the feasibility of STED super-resolution imaging of human FFPE skin tissue sections. In combination with deconvolution, this improves the fluorescence signals and suppresses the background noise. Furthermore, this pipeline was used to quantitatively evaluate the spatial relationships between the NS marker SON and PI(4,5)P2.

### 2.2 Quantitative STED microscopy reveals the nanoscale spatial relationship between nPI(4,5)P2 and the NS marker SON in human keratinocytes

**FIGURE 2:**
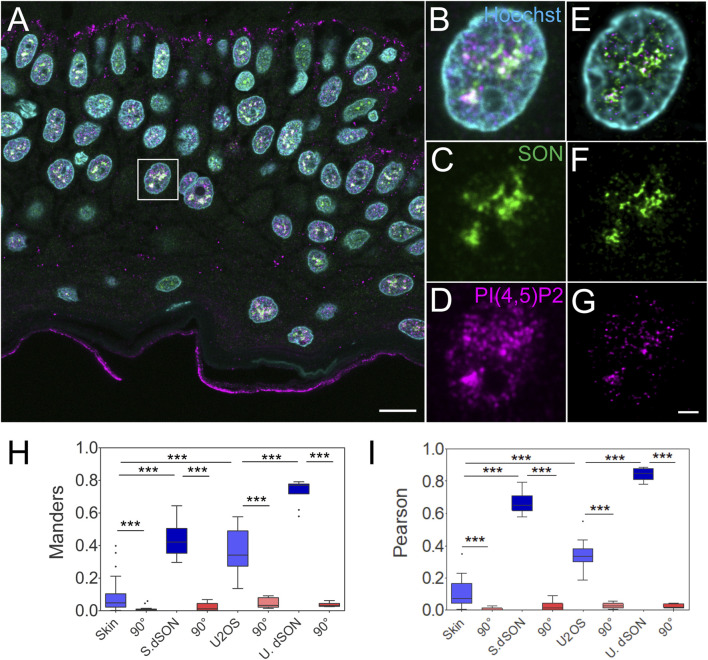
Quantitative image analysis of nuclear antigens in human FFPE skin sections. Overview of the sample **(A)**, zoomed in to the boxed region **(B–G)**, confocal **(B–D)** and STED with deconvolution **(E–G)** images of the same nucleus with Hoechst nuclear stain in cyan, SON in green and PI(4,5)P2 in magenta. Scale bars 5 µm. Tukey plots of Manders overlap **(H)** and Pearson correlation **(I)** coefficients between PI(4,5)P2 and SON in the skin or U2OS nuclei, and between double labelled SON in the skin (S. dSON) or U2OS (U. dSON) nuclei and corresponding rotated images (90°).

The majority of nPI(4,5)P2 localizes to NS in cultured human cell lines when visualzied by immunofluorescence and microscopy (Boronenkov et al., 1998; Mellman et al., 2008; Mellman and Anderson, 2009; Sobol et al., 2018; Hoboth et al., 2021a; Balaban et al., 2021; Hoboth et al., 2021b; Hoboth et al., 2021c). However, the distribution of PI(4,5)P2 within the nuclei of human tissues is unknown. Therefore, we quantitatively evaluated the spatial relationship between the nPI(4,5)P2 and the NS marker SON in the individual keratinocyte nuclei in human FFPE skin sections (Figure 2A). We acquired STED Z-stacks encompassing individual keratinocyte nuclei and subjected them to deconvolution. A comparison between confocal (Figures 2B–D) and deconvolved STED images (Figures 2E–G) showed improved resolution, which is critical for precise quantification of the acquired signals. We measured the Manders overlap (Figure 2H) and Spearman rank correlation (Figure 2I) between nuclear PI(4,5)P2 and SON. We calculated these coefficients using JaCoP (Bolte and Cordelieres, 2006) ImageJ2 (Rueden et al., 2017) plugin and compared them between the real images and images in which one channel was rotated 90° with respect to the second channel (Dunn et al., 2011; Hoboth et al., 2021b; Hoboth et al., 2021c; Noordstra et al., 2022). Moreover, we also measured Manders and Pearson coefficients in the deconvolved STED Z-stacks of individual keratinocyte nuclei of the FFPE tissue sections that were immunolabeled only with one primary antibody, against NS marker SON, and two secondary antibodies against the anti-SON antibody, conjugated with either Abberior Star 580 or Abberior Star 635P (Supplementary Figure S2). This procedure allowed us to find the best experimentally achievable degree of overlap and correlation between the two signals (Hoboth et al., 2021c). For the comparison, we also measured the PI(4,5)P2-to-SON Manders (Figure 2H) and Pearson (Figure 2I) coefficients in the deconvolved STED Z-stacks of the individual nuclei of fixed U2OS cells cultured in a monolayer (Supplementary Figures S1A–D). Finally, we immunofluorescently stained cultured U2OS cells with two secondary antibodies (one conjugated with Abberior Star 580 and other with Abberior Star 635P) against only the anti-SON primary antibody (Supplementary Figures S1E–H). Thereby, we measured the experimentally best achievable Manders overlap and Pearson correlation coefficients in the fixed monolayer U2OS cell nuclei.

Manders (M) overlap (0.08 ± 0.02; N = 4; n = 37) and Pearson (P) correlation (0.1 ± 0.01; N = 4; n = 37) coefficients between PI(4,5)P2-635 and SON-580 in the skin were significantly reduced (to 0.008 ± 0.002 and 0.006 ± 0.001 resp., both *p* < 0.005) by rotating first channel 90° with respect to the second channel (Figures 2H,I). This indicates a specific, although low, overlap and correlation, between PI(4,5)P2 and SON in the keratinocyte nuclei. The M and P coefficients for the double-labeled SON in skin (S. dSON) indicated the best experimentally achievable overlap and correlation, as well as the super-resolved separation of the signal of two fluorophores conjugated to two physically distinct secondary antibodies. The M and P PI(4,5)P2-to-SON coefficients were 3-4 fold higher in U2OS cells cultured in a monolayer and reached the highest values among all evaluated data sets in the monolayer cultured U2OS cells in which SON was double-labelled with two fluorophores. In U2OS cells, both coefficients were also significantly reduced by rotating the first channel 90° with respect to the second channel, indicating specific co-patterning of nPI(4,5)P2 with SON.

Taken together, we measured the specific spatial relationship between nPI(4,5)P2 and the NS marker SON in human FFPE skin tissue sections. Moreover, we compared it with the spatial relationship between nuclear PI(4,5)P2 and the NS marker SON in the U2OS cell line that we previously characterized by dSTORM (Hoboth et al., 2021b; Hoboth et al., 2021c). Higher M and P in cultured U2OS could be either due to the biological difference between different cell types or due to the processing of the FFPE tissue sections. Therefore, we further tested how the FFPE process affected the spatial co-patterning between PI(4,5)P2 and SON in cultured U2OS cells.

### 2.3 Processing of the samples affects the nanoscale patterning of the nuclear antigens

**FIGURE 3:**
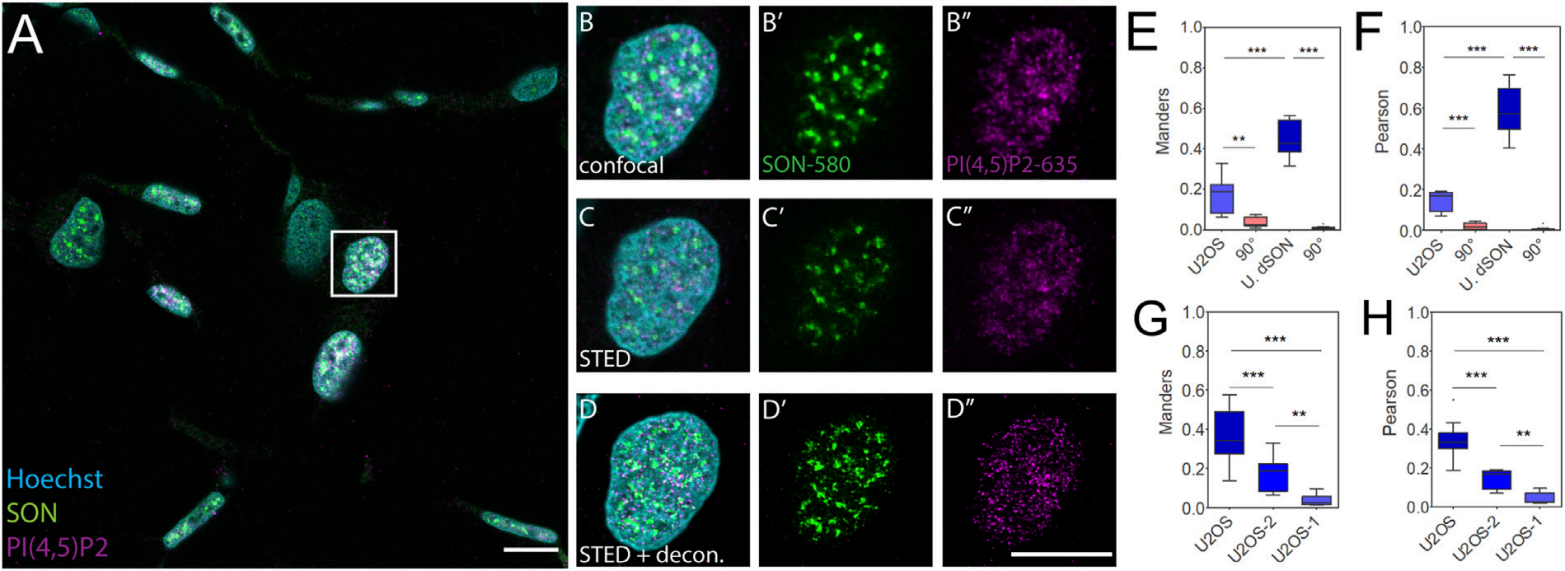
Control quantitative imaging of the nuclear antigens in FFPE U2OS cells. Overview of the sample **(A)**, zoomed in to the boxed region **(B-D**″**)**, confocal **(B-B**″**)** and STED **(C-C**″**)** with deconvolution **(D-D**″**)** images of the same nucleus with Hoechst nuclear stain in cyan, SON in green and PI(4,5)P2 in magenta. Scale bars 5 µm. Tukey plots of Manders overlap **(E)** and Pearson correlation **(F)** coefficients between PI(4,5)P2 and SON in FFPE U2OS nuclei or between double labelled SON in the FFPE U2OS nuclei (U. dSON) and corresponding rotated images (90°). Tukey plots of Manders overlap **(G)** and Pearson correlation **(H)** coefficients between PI(4,5)P2 and SON in U2OS cells imaged in monolayer (U2OS), U2OS cells processed according to smethod 2 (U2OS-2) or method 1 (U2OS-1).

Given the differences between FFPE tissue sections and U2OS cells cultured in a monolayer even in the SON double-labeled controls, we investigated whether this was due to cell type differences or if FFPE processing affected the co-patterning of nPI(4,5)P2 with SON. To this end, we sectioned FFPE U2OS cells (4 µm sections) pre-processed in two slightly different ways and stained the sections using the same procedure as that for FFPE skin tissue sections. In the first pre-processing procedure (Supplementary Figure S3), we first trypsinized and pelleted the U2OS cells cultured in a monolayer (pre-processing method 1; U2OS-1). We then fixed the cell pellet and embedded it in low melting agarose. We further processed the sample as tissue for paraffin embedding. In the second procedure (Figure 3), we first fixed the U2OS cells in a monolayer, mechanically removed them from the culture surface and pelleted them (pre-processing method 2; U2OS-2). Next, we embedded the cell pellet in low melting agarose and then in paraffin.

We first captured the confocal overview of a section of FFPE U2OS-1 cell pellet immunofluorescently labeled for the NS markers SON-580 and PI(4,5)P2-635 (Supplementary Figure S3A). Then we imaged individual nuclei from this sample by confocal microscopy (Supplementary Figure S3B–B″). Next, we collected the STED (Supplementary Figure S3C–C″) Z-stacks of these nuclei and subjected these data to deconvolution (Supplementary Figure S3D–D″). In contrast to the keratinocyte nuclei in the skin tissue (Figure 1; Figure 2) and similarly to the nuclei of U2OS cells imaged on the glass surface in a monolayer (Supplementary Figure S1), the nuclei of either U2OS-1 (Supplementary Figure S3) or U2OS-2 (Figure 3) pre-processed cells were wider and thinner even in the FFPE pellet. This morphological feature of U2OS cells, which is, at least in part, due to the flattening of the nuclei in the cells cultured in a monolayer on the coverslip surface, was preserved during sample preparation. Fixation of the cells after the pelleting (U2OS-1) resulted in the very low M (0.04 ± 0.007) and P (0.05 ± 0.007) coefficients of the overlap and correlations, between PI(4,5)P2 and SON (Supplementary Figures S3E, F). This was presumably due to the disruption of cell morphology by removal of the living cells from the culture surface, which affects even the nuclear antigens, especially at the nanoscale uncovered by SRM. Processing of the U2OS cells fixed in the monolayer and then pelleted (U2OS-2) better preserved the nanoscale sub-nuclear organization, as documented by higher M (0.18 ± 0.03) and P (0.15 ± 0.02) coefficients (Figures 3E,F) compared to the U2OS-1 sample (Figures 3G,H). Nevertheless, PI(4,5)P2-to-SON M overlap and P correlation coefficients in U2OS-2 sample were lower compared to the U2OS cells fixed, stained and imaged in the monolayer, without paraffin embedding (Figures 3G,H). This suggested that paraffin embedding and/or sectioning and processing of the FFPE sections affects the sub-nuclear architecture, at least when quantitatively analyzed at nanoscale by SRM. Moreover, the M (0.51 ± 0.04) and P (0.72 ± 0.04) coefficients for the double-labelled SON, indicating the best experimentally measurable overlap and correlation, were only slightly (but significantly; both *p* < 0.05) lower than the M (0.75 ± 0.02) and P (0.84 ± 0.01) coefficients for the double-labelled SON in U2OS cells grown in monolayer cultures. The approx. half reduction of PI(4,5)P2-SON M and P coefficients between the U2OS-2 sample compared with the less extensively processed flat U2OS cells suggests that the SON protein antigen is better preserved during FFPE sample processing than the lipid PI(4,5)P2 antigen. Taken together, the quantitative comparison of the spatial co-patterning between PI(4,5)P2 and SON in FFPE U2OS cells and flat U2OS cells indicated that paraffin embedding, sectioning and subsequent processing of the sections before immunolabeling mildly but significantly affected the nanoscale organization of the nuclear antigens. This indicates that the differences measured between keratinocytes (Figures 2H,I) and U2OS cells are not solely cell type specific but are also introduced by sample preparation. Next, we quantitatively evaluated the spatial co-patterning between PI(4,5)P2 and SON in human FFPE warts sections and compared them with skin sections that were processed in the exactly same fashion.

### 2.4 Nuclear PI(4,5)P2 differs between normal skin and skin warts

**FIGURE 4:**
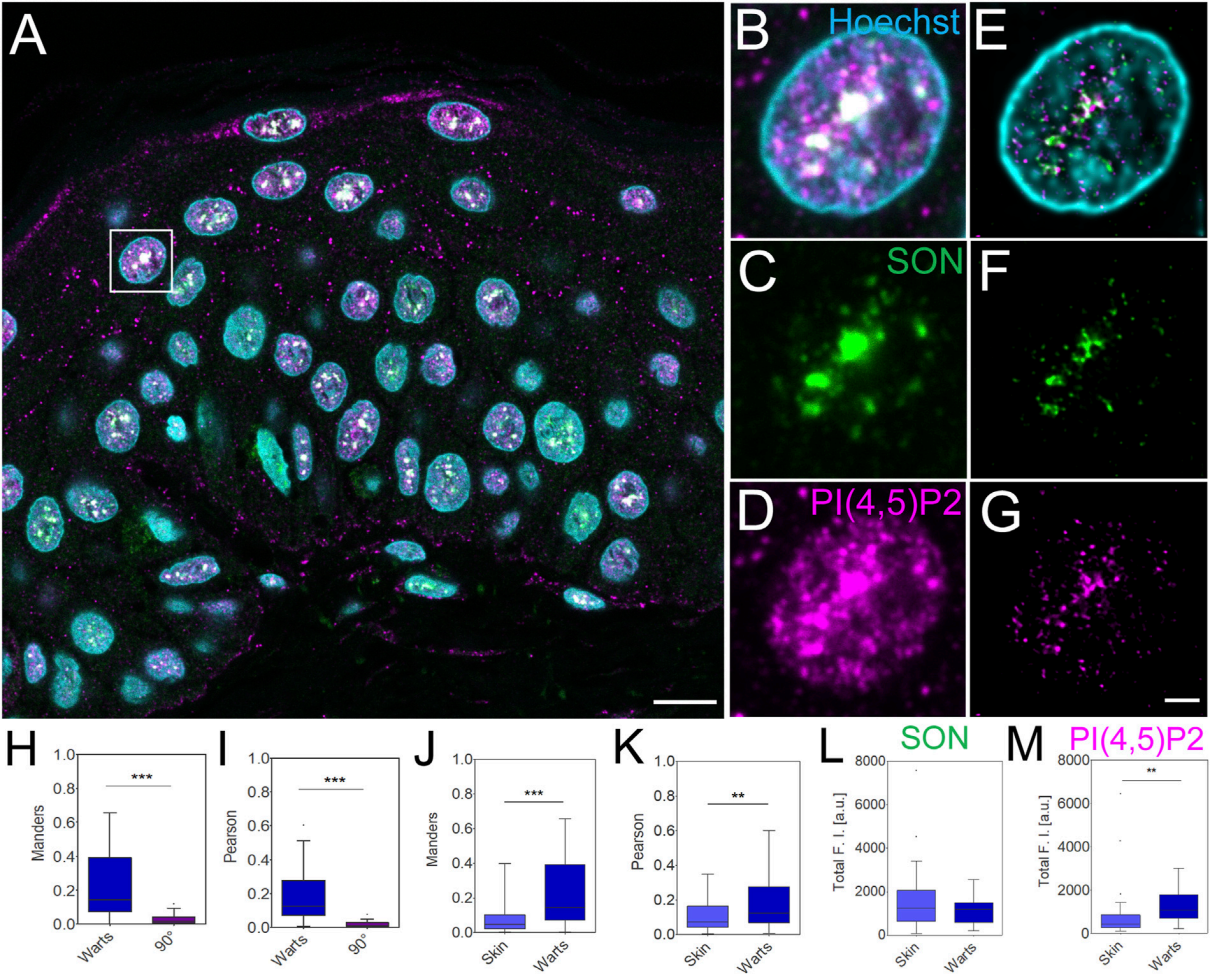
Imaging and quantification of the nuclear antigens in human FFPE warts sections. Overview of the sample **(A)**, zoom in to the boxed region **(B–G)**, confocal **(B–D)** and STED with deconvolution **(E–G)** images of the same nucleus with Hoechst nuclear stain in cyan, SON in green and PI(4,5)P2 in magenta. Scale bar 5 µm **(A)** or 1 µm **(B–G)**. Tukey plots of Manders overlap **(H and J)** and Pearson correlation **(I and K)** coefficients between PI(4,5)P2 and SON in wart nuclei and corresponding 90° rotated images **(H and I)** and comparison between skin and wart nuclei **(J and K)**. Total SON **(L)** and PI(4,5)P2 **(M)** fluorescence intensity **(F, I)** in arbitrary units (a.u.) in skin and wart nuclei. Quantitative measurements for skin nuclei are in **(J and K)** the same as in Figures 2H,I.

Increased levels of immunofluorescently labelled nPI(4,5)P2 in SCC have previously been linked to infections with oncogenic HPV (Marx et al., 2018). Nevertheless, the precise sub-nuclear localization of nPI(4,5)P2 was hindered due to the resolution limit of conventional microscopy. Benign HPV infection causes warts (Loo and Tang, 2014; Howley and Pfister, 2015; Hufbauer and Akgül, 2017), which can be removed by minimal surgical excision. Here we used STED microscopy to image and quantify the overlap and correlation between nPI(4,5)P2 and SON as well as the immunofluorescently labelled nPI(4,5)P2 and SON levels in human clinical FFPE warts sections and compare it with normal human skin (Figure 4).

Human skin warts were surgically removed and processed by FFPE as the above-analyzed skin tissue. FFPE warts were then sectioned into 4 µm sections and dewaxed prior to indirect immunofluorescence staining. Dewaxed wart sections were immunolabeled with primary antibody against SON recognized by secondary antibody conjugated with Abberior Star 580, and primary antibody against nPI(4,5)P2 and secondary antibody conjugated with Abberior Star 635P (Figures 4A–G). Nuclei were labeled by Hoechst-JF503. Figure 4A shows an overview of the wart section and Figures 4B–D individual nuclei were imaged by confocal microscopy. A comparison of the confocal images (Figures 4B–D) with deconvolved STED nanographs of the same nuclei (Figures 4E–G) documented the feasibility of the multi-color SRM on another clinically relevant sample, human warts, in addition to the samples of normal human skin (Figure 1; Figure 2). Similarly to normal skin, in skin warts the nPI(4,5)P2 (Figures 4E,G) displayed signal that mostly overlapped with the SON signal (Figures 4E,G), but was also present in the nucleoplasm (Figures 4E,G).

In the deconvolved STED Z-stacks of individual nuclei from the warts, we measured the M overlap (Figure 4H) and P correlation (Figure 4I) between nPI(4,5)P2 and SON. We compared M and P between the real images and images in which one channel was rotated 90° with respect to the second channel. Significant reduction of both coefficients by rotating one channel in the dual-color images documented the specific overlap and correlation (Dunn et al., 2011; Hoboth et al., 2021b; Hoboth et al., 2021c; Noordstra et al., 2022) of nPI(4,5)P2 with SON in FFPE wart sections. Next, we compared the overlap and correlation of nPI(4,5)P2 with SON in the FFPE skin sections (Figure 2) with the wart sections (Figure 4). We measured significantly higher M (Figure 4J) and P (Figure 4K) in the nuclei in warts compared with the nuclei in skin. Finally, we measured the total SON-580 (Figure 4L) and PI(4,5)P2-635 (Figure 4M) fluorescence intensity (FI) and compared it between skin and wart nuclei. The total SON-580 FI did not significantly differ between skin and wart nuclei (Figure 4L), but the total PI(4,5)P2-635 FI (Figure 4M) was significantly higher in wart nuclei (Figure 4G) compared to skin nuclei (Figure 2G). This was consistent with the previous link between HPV infection and increased nPI(4,5)P2 staining (Marx et al., 2018). It also indicated that higher M overlap (Figure 4J) and P correlation (Figure 4K) between PI(4,5)P2 and SON is due to the increased nPI(4,5)P2 levels in warts nuclei and not due to the changes in the SON levels. Increased total nPI(4,5)P2 FI together with unchanged total SON FI, which resulted in the increased Manders coefficient, indicates that skin contain more nPI(4,5)P2 staining within SON^+^ NS compared to healthy skin.

In summary, STED nanoscopy combined with the deconvolution allowed us to quantitatively characterize the specific nanoscale co-patterning between nPI(4,5)P2 and SON in two different clinically relevant samples, normal skin and skin warts. Moreover, we quantitatively showed a higher overlap and correlation of nPI(4,5)P2 with SON in warts than in normal skin. Our data document the feasibility of the nanoscale analysis of the nuclear antigens in human clinical FFPE tissue sections, providing detailed information about nPI(4,5)P2 in human tissues and shows the differences between healthy and HPV-infected skin, which has potential clinical relevance.

## 3 Discussion

In this study, we present a quantitative multicolor SRM analysis of the functional nuclear architecture in human clinical FFPE tissue sections. The past decade has witnessed a rapid progress in the biology of the cell nucleus and increasing understanding of its functional organization in particular, owing to the progressive development and application of SRM techniques (Schermelleh et al., 2019; Hoboth et al., 2021a; Lelek et al., 2021). Nevertheless, many physiological and pathophysiological processes, such as viral infections, cellular transformation and oncogenesis, remain relatively unexplored, particularly in the context of tissues and organisms. This scarcity of data is even more prominent in human samples. Here, we revealed tissue-specific nuclear architecture and quantitatively assessed the differences between nuclei in human skin and warts at the nanoscale.

SRM studies of subcellular architecture at the nanoscale started with single-color STED imaging of cultured cells (Willig et al., 2006). The nanoscale tissue architecture was initially studied by single-color STED microscopy of dendritic spines in living organotypic slices from the mouse hippocampus (Nagerl et al., 2008) and then in the living mouse brain (Berning et al., 2012). Later, STED supplemented EM and biochemical analyses to render a high-resolution atlas of isolated synaptic boutons (Wilhelm et al., 2014). Multicolor SMLM provided further quantitative insights into the molecular architecture of chemical synapses in mouse brain cryo-sections (Dani et al., 2010). These studies were, however, limited to animal models. SRM studies of human samples involved cryo-preserved samples (Codron et al., 2021; Hernandez et al., 2022). However, FFPE represents the most practical way to archive and manipulate clinical samples (Kokkat et al., 2013; Lou et al., 2014). Indeed, various biobanks contain a vast amount of human tissues resected in clinical procedures and therefore provide a valuable, yet untapped source of healthy and diseased human tissues (Baker, 2012; Eiseman and Haga, 1999). Earlier single-color STED microscopy of human FFPE rectal cancer tissue revealed details of the mitochondrial architecture that was previously hindered by the diffraction barrier (Ilgen et al., 2014). Single-color SMLM in human FFPE breast cancer tissue allowed insights into the nanoscale organization of the cell membrane marker HER2, outer mitochondrial membrane protein TOM20 and Lamin B1, a component of the nuclear envelope (Creech et al., 2017). Nevertheless, single-color SRM allowed neither to study multiple markers simultaneously, nor to evaluate their mutual spatial relationships. Therefore, here we implemented multicolor STED imaging of human FFPE tissue sections followed by the image deconvolution and quantitative image analysis. The FFPE samples used in our study were 4–5 years old, which is consistent with previous findings demonstrating the suitability of STED super-resolution microscopy for revealing nanoscale protein distributions in tissues stored for decades in biorepositories (Ilgen et al., 2014). This pipeline is adaptable for the various quantitative analyses of the molecular signatures linked to various human physiological as well as pathophysiological conditions and thus allows to study the nanoscale details in clinically relevant FFPE specimens stored for years in various biobanks as a pretext for future personalized medicine (Hewitt, 2011). Here we used it to quantitatively analyze the nanoscale spatial relationship between nPI(4,5)P2 and the NS marker SON in normal human skin and skin warts.

Previous visualizations of NS in tissues used diffraction-limited confocal microscopy (George-Tellez et al., 2002; Saitoh et al., 2012; Kuga et al., 2016). This technique allowed neither the precise separation between individual NS nor gained insight into the detailed organization of individual NS. Super-resolution is hence critical for investigating the localization of nPI(4,5)P2 within the SON matrix (Hoboth et al., 2021b; Hoboth et al., 2021c). The STED nanoscopy followed by image deconvolution that we applied here allowed us to quantitatively evaluate the co-patterning of nPI(4,5)P2 with SON at the nanoscale context in healthy skin and skin warts, caused by HPV infection. We super-resolved here for the first time within the context of human clinical FFPE tissue the multiple nuclear antigens, specifically the molecular anatomy of NS and its associated nPI(4,5)P2 pool. Nanoscale protein and lipid interactions execute specific cellular functions and their impairment or hijacking by pathogens leads to the development and progression of disease (Rattay et al., 2023). Here, we quantitatively showed that, compared with healthy skin nuclei, the staining of SON^+^ NS-associated pool of nPI(4,5)P2 is increased in nuclei in HPV-induced wart. We have thus shown that elevated nPI(4,5)P2 staining is not only found in HPV-associated cancers (Marx et al., 2018), but also in benign skin lesions induced by low-risk HPV. Hence, one can envision that it is clinically important to gain quantitative insights into the nanoscale organization of human tissues in both healthy and diseased states. Our data presented here and the further application of quantitative SRM will help us to understand the nanoscale molecular organization associated with physiological as well as pathophysiological processes, including but not limited to virus infection, cellular transformation or oncogenesis.

## 4 Material and methods

### 4.1 Ethics statement

The collection and analysis of FFPE skin and warts sections was approved by the local ethics-committee at the Department of Pathology, University of Cologne, Germany. Written informed consent was obtained from all patients in accordance with the Declaration of Helsinki. For biopsy materials from archival paraffin blocks of human skin and warts informed consent was obtained from all the subjects and ethical approval obtained from the Ethics Committee at the University of Cologne. FFPE samples were collected between years 2018-2019 and thus archived for 4–5 years before analysis presented in this study.

### 4.2 Cell cultures

U2OS cells were grown in DMEM with 10% FBS at 37°C and 5% CO_2_. Cells were plated 1 day before staining in ~ 50% confluence on the high-precision 12 mm round coverslips with 1.5H thickness (Marienfeld 0107222).

### 4.3 Paraffin embedding and sectioning of cultured cells

U2OS cells were grown as above but to confluence in T-175 flasks. Cells in one flask (U2OS-1) were washed twice with PBS, removed by trypsinization in 0.05% trypsin in PBS with EDTA for 5 min at 37°C, washed off in 15 mL PBS into the centrifugation tube, pelleted for 5 min at 1,000 x *g*, washed and pelleted twice with PBS for 5 min at 1,000 x *g*. Pellets were fixed by resuspending in 0.5 mL of 2% PFA for 30 min at RT, then washed and pelleted three times with 5 mL of PBS for 5 min at 1,000 x *g*. Supernatant was aspirated and fixed cell pellet was embedded in 1% low melting agarose dissolved at 37°C (cells:agarose 1:4). Cells in second flask (U2OS-2) were rinsed twice by PBS and then fixed in flask by 2% PFA for 30 min at RT, washed three times with PBS, scraped the cells in 15 mL PBS, transferred into the centrifugation tube and pellet for 5 min at 1,000 x *g*. Supernatant was then removed and cells were embedded in 1% low melting agarose dissolved at 37°C (cells:agarose 1:4). Both agarose-embedded fixed cell pellets were dehydrated and penetrated with wax on automated tissue processor Leica ASP200S and blocks were created using Leica, E.G.,1150H paraffin embedding station with the following program: 70% EtOH 45°C 2 × 30 min, 95% EtOH 2 × 30 min, 1 × 60 min, 1 × 90 min; 100% xylene at 45°C 2 × 45 min, 1 × 90 min; 100% paraffin at 65°C 2 × 60 min, 1 × 80 min.

### 4.4 Sectioning of U2OS cells

Paraffin blocks were cooled for few minutes in the fridge/freezer before cutting. U2OS-1 and U2OS-2 FFPE samples were cut into 4 µm sections on Microtome Leica RM2255 and collected on polylysined slides with the water bath heated to 42°C. Sections were removed from water bath, placed on polylysined slides and baked o/n at 42°C. Slides were prepared as follows: washed by 96% EtOH, air-dried, incubated in 0.01% poly-L-Lysine for 10 min and then baked for 1h at 60°C or air dried at RT o/n.

### 4.5 Immunohistochemistry

FFPE sections were dewaxed by following washes: xylene: twice 4 min; xylene 1:1 with 100% EtOH 4 min; 96% EtOH: 2 × 4 min; 90% EtOH 4 min; 70% EtOH 4 min; 50% EtOH 4 min; rinse in cold water. Sections were incubated in 0.1% Triton X-100 in PBS for 20 min, washed 3-times for 5 min by PBS and blocked in filtered 5% BSA in PBS for 30 min. Cells were incubated for 60 min with primary antibodies diluted in 5% BSA in PBS, washed 3-times for 5 min in PBS and incubated for 40 min with secondary antibodies diluted in 5% BSA in PBS. Then the cells were incubated with 1 mM Hoechst-JF503 (Janelia Farm) in PBS for 5 min, washed twice 5 min in PBS, dip in ddH2O, air-dry at RT and mount coverslips on microscopy glass in 5 uL of 90% glycerol with 4% n-Propyl gallate (Sigma).

### 4.6 Immunocytochemistry

The cells were washed twice with PBS (pH 7.4) and fixed for 30 min in 2% PFA in PBS, washed 3-times for 5 min with PBS, then permeabilized in 0.1% Triton X-100 in PBS for 20 min, washed 3-times for 5 min by PBS and blocked in filtered 5% BSA in PBS for 30 min. Cells were incubated for 45 min with primary antibodies diluted in 5% BSA in PBS, washed 3-times for 5 min in PBS and incubated for 30 min with secondary antibodies diluted in 5% BSA in PBS. Then the cells were incubated with 1 mM Hoechst-JF503 (Janelia Farm) in PBS for 5 min, washed twice 5 min in PBS, dip in ddH2O, air-dry at RT and mount coverslips on high-precission 1.5H square 18 × 18 mm coverslips glass (Zeiss) in 5 uL of NPG mounting media.

### 4.7 Antibodies

Following primary antibodies and concentrations were used: mouse ascites IgM anti-PI(4,5)P2 2C11 (Z-A045; Echelon Biosci. Inc., United States) 5 μg/mL; rabbit polyclonal IgG anti-SON (ab121759; Abcam, United Kingdom) 1 μg/mL. Following secondary antibodies and concentrations were used: goat anti-mouse IgG Abberior STAR 635P (ST635P-1001-500UG; Abberior) 10 μg/mL; goat anti-rabbit IgG Abberior STAR 635P (ST635P-1002-500UG; Abberior) 10 μg/mL; goat anti-mouse IgG Abberior STAR 580 (ST580-1002-500UG; Abberior) 10 μg/mL.

### 4.8 Confocal and STED microscopy

Imaging was performed on Leica TCS SP8 STED 3x inverted DMi8 microscope with pulsed white light laser 470–640 nm 1.5 mW and 775 nm pulse STED laser >1.5 W controlled by Leica Application Suite X software and equipped with HC PL APO CS2 100x/1.40 OIL objective used with Leica Type F immersion oil n = 1.518. Unidirectional xyz scanning speed was 400 Hz, line accumulation 6 for Hoechst-JF503 and 8 for SON-580 or PI(4,5)P2-635. Pixel size 20 nm in X and Y for STED and 30 nm for confocal and 100 nm in Z. Channel 1 (Hoechst-JF503): 10% 503 nm laser; 10% 503 nm laser; PMT Gain 700. Channel 2 (Abberior STAR 580): 10% 585 nm laser; 775 Notch filter; 80% 775 nm STED laser, 30% 3D STED; Hybrid detector (HyD) 589–616 nm, photon-counting mode, gain 100, gating 0.4–10 ns. Channel 3 (Abberior STAR 635P): 7% 633 nm laser; 775 Notch filter; 50% 775 nm STED laser; 30% 3D STED; HyD 639–698 nm, photon-counting mode, gain 100, gating 0.3–10 ns. Sequential scanning; STED laser off for confocal.

### 4.9 Deconvolution

Z-stacks of STED images were deconvolved using Huygens Professional 22.10 software (Scientific Imaging B.V.). Data sets were processed using Workflow Processor. The workflow consisted of selecting images, setting up the microscopy and deconvolution parameters and saving deconvolved images as 8-bit TIFF single files for individual channels (which were later used for the quantitative analyses; see below). Microscopy parameters were optimized and set as follows. Sampling intervals were ≤20 nm in X and Y and ≤20 nm in Z. Numerical aperture was 1.4; refractive indexes of the lens immersion oil was 1.518 and of the embedding media 1.458; objective quality was good, coverslip position was 0 µm and imaging direction was downward. For STED channel 1, which corresponded to SON-580 the backprojected pinhole was 195 nm; excitation (ex.) and emission (em.) wavelengths (*λ*) were 585 and 602 nm, resp., ex. fill factor 2. STED depletion mode was pulsed, saturation factor 20, STED *λ* = 775, STED immunity factor 10 and STED 3X was 30%. For STED channel 2, which corresponded to PI(4,5)P2-635 were all parameters the same, with the following exceptions. Backprojected pinhole was 216 nm; ex. and em. *λ* were 633 and 651 nm, resp. and STED saturation factor 25. Classic MLE algorithm with stabilization of Z-slices was used for all channels with the following specifications for Hoechst-JF503 channel: theoretical PSF mode; max. iterations 20; optimized iteration mode; 0.01% quality change threshold; 3.9 signal-to-noise ratio; acuity mode on; background mode—lowest value; background estimation radius 0.7; relative background 0. Specifications that differed for SON-580 or PI(4,5)P2 channels from Hoechst-JF503 channel: max. iterations 15; signal-to-noise ratio was 4 for SON-580 and 5.1 for PI(4,5)P2.

### 4.10 Image analyses

All quantifications were performed on deconvolved STED images. Fluorescence intensity (FI) profiles, pseudo-coloring, Fire LUT assignment, image post-processing for figures, FI measurements and one-channel rotation were done in ImageJ2 (Rueden et al., 2017). Manders and Pearson coefficients were calculated using JaCoP plug-in (Bolte and Cordelieres, 2006) for individual planes of a Z-stack after Moments auto-threshold was applied on deconvolved STED Z-stacks. Images were batch processed using self-written macro. Figures were created in Adobe Illustrator.

### 4.11 Graphs and statistics

Tukey whiskers plots and statistical evaluations were done in Prism (GraphPad). Paired, one-tailed *t*-test was used for the comparison between real and rotated images. Mann-Whitney test was used for comparison between various samples. Statistical significance: *p < 0.05; **p < 0.01; ***p < 0.005. We measured 32 skin nuclei in 5 independent stainings of sections from two independent FFPE tissue blocks and 27 warts nuclei nuclei in 5 independent stainings of sections from two independent FFPE tissue blocks.

## Data Availability

The datasets presented in this study can be found in online repositories. The names of the repository/repositories and accession number(s)/DOI: 10.5281/zenodo.8060866 and 10.5281/zenodo.8060840 can be found here: https://zenodo.org/record/8060840 and https://zenodo.org/record/8060866.
