# Supplementary material for: Quantitative super-resolution microscopy reveals the differences in the nanoscale distribution of nuclear phosphatidylinositol 4,5-bisphosphate in human healthy skin and skin warts
Source: Front Cell Dev Biol. 2023 Jul 7;11:1217637. doi: 10.3389/fcell.2023.1217637 (PMC10361526; doi:10.3389/fcell.2023.1217637)
Supplement: Supplementary file 1 [file DataSheet1.pdf]

<u>Supplementary figures</u> for

Hoboth P, Sztacho M, Quaas A, Akgül B and Hozák P (2023), Quantitative superresolution microscopy reveals the differences in the nanoscale distribution of nuclear phosphatidylinositol 4,5-bisphosphate in human healthy skin and skin warts. Front. Cell Dev. Biol. 11:1217637

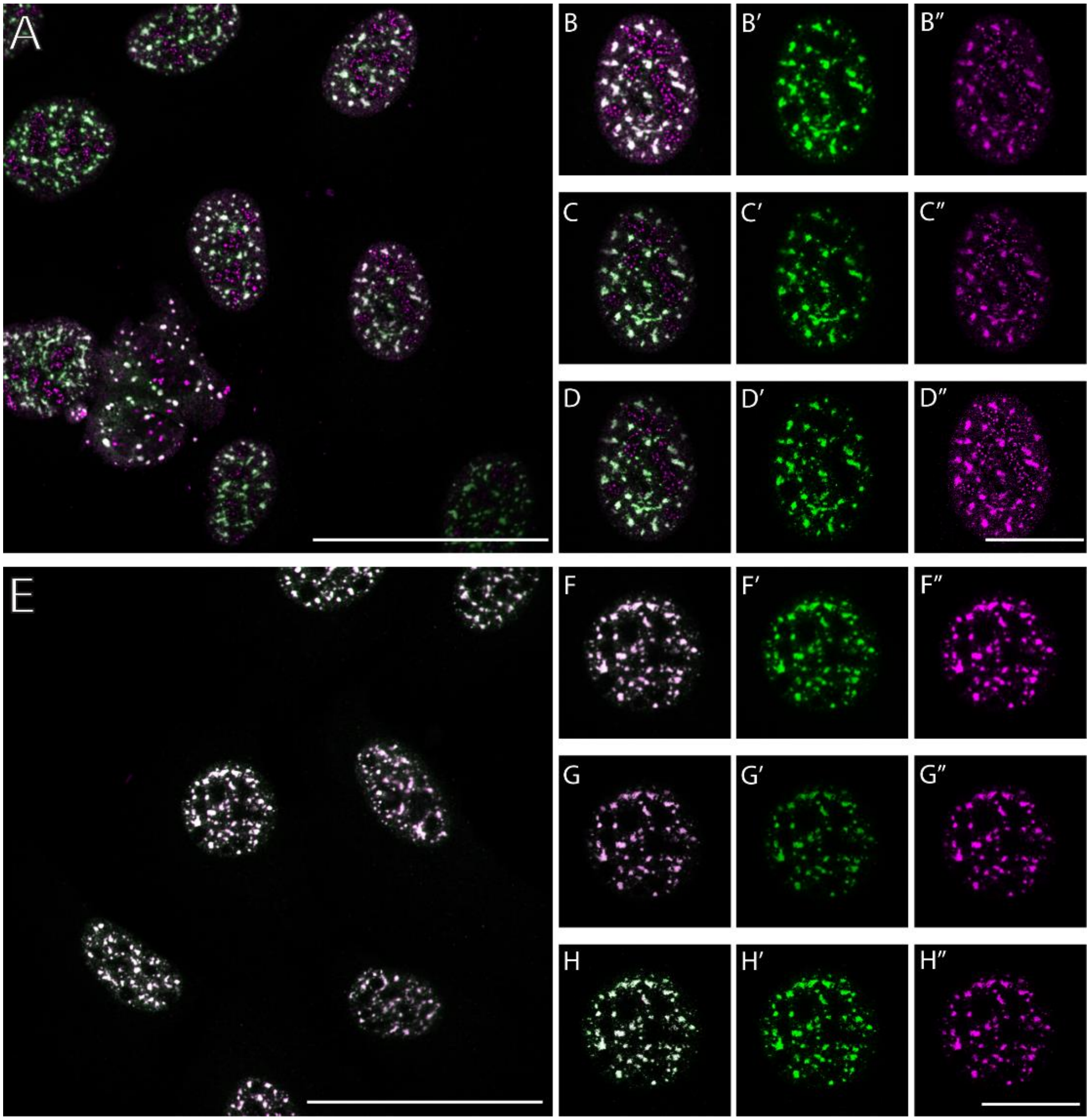

Supplementary figure 1. Imaging of cultured U2OS cells. Overview of the U2OS cells monolayer grown on microscopy slide (A), zoom in to a nucleus (B-D''), confocal (B-B'') and STED (C-C'') with deconvolution (D-D'') images merged (B, C, D), SON in green (B', C', D') and PI(4,5)P2 in magenta (B'', C'', D''). SON double-labelled U2OS cell monolayer overview (E), zoom in to a nucleus (F-H''), confocal (F-F'') and STED (G-G'') with deconvolution (H-H'') images merged (F, G, H), SON-580 in green (F, F', G, G', H, H') and 635P in magenta (F, F'', G, G'', H, H''). Scale bars (A, E) 50 µm (D'', H'') 10 µm.

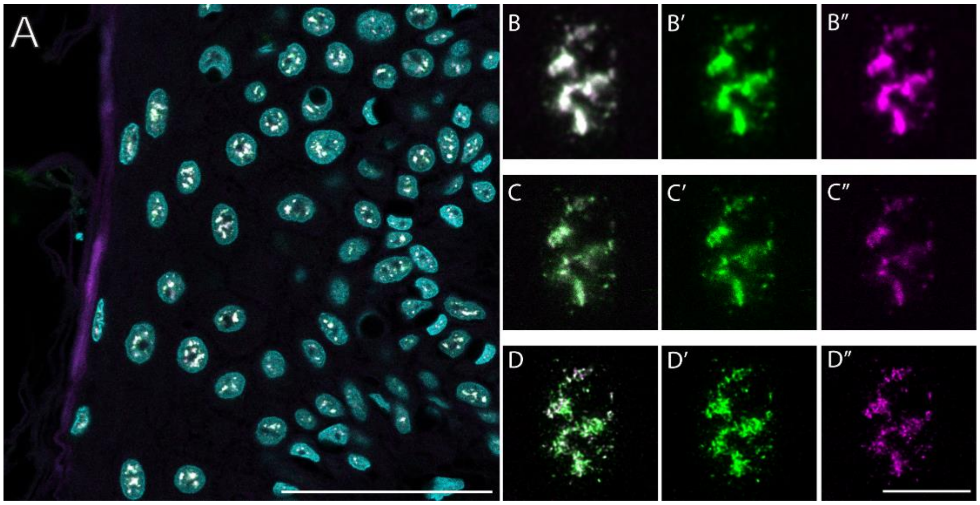

Supplementary figure 2. Imaging of the double-labelled SON in FFPE skin section. Overview with Hoechst nuclear stain in cyan and SON in green and magenta (A), zoom in to a nucleus (B-B''), confocal (B-D) and STED with deconvolution (E-G) images of the same nucleus, SON. Zoom in to a nucleus (B-D''), confocal (B-B'') and STED (C-C'') with deconvolution (D-D'') images merged (B, C, D), SON-580 in green (B, B', C, C', D, D') and 635P in magenta (B, B'', C, C'', D, D''). Scale bars (A) 10 µm (D'') 5 µm.

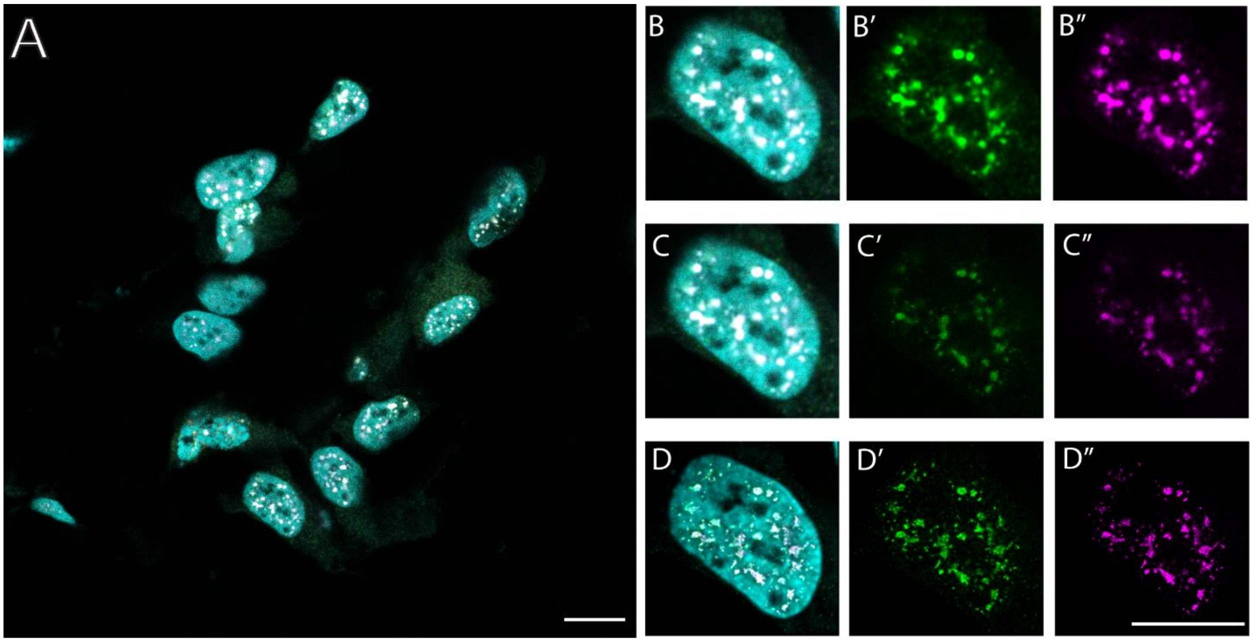

Supplementary figure 3. Imaging of the double-labelled SON in FFPE U2OS-2 section. Overview with Hoechst nuclear stain in cyan and SON in green and magenta (A), zoom in to a nucleus (B-B''), confocal (B-D) and STED with deconvolution (E-G) images of the same nucleus, SON. Zoom in to a nucleus (B-D''), confocal (B-B'') and STED (C-C'') with deconvolution (D-D'') images merged (B, C, D), SON-580 in green (B, B', C, C', D, D') and 635P in magenta (B, B'', C, C'', D, D''). Scale bars (A) 10 µm (D'') 5 µm.

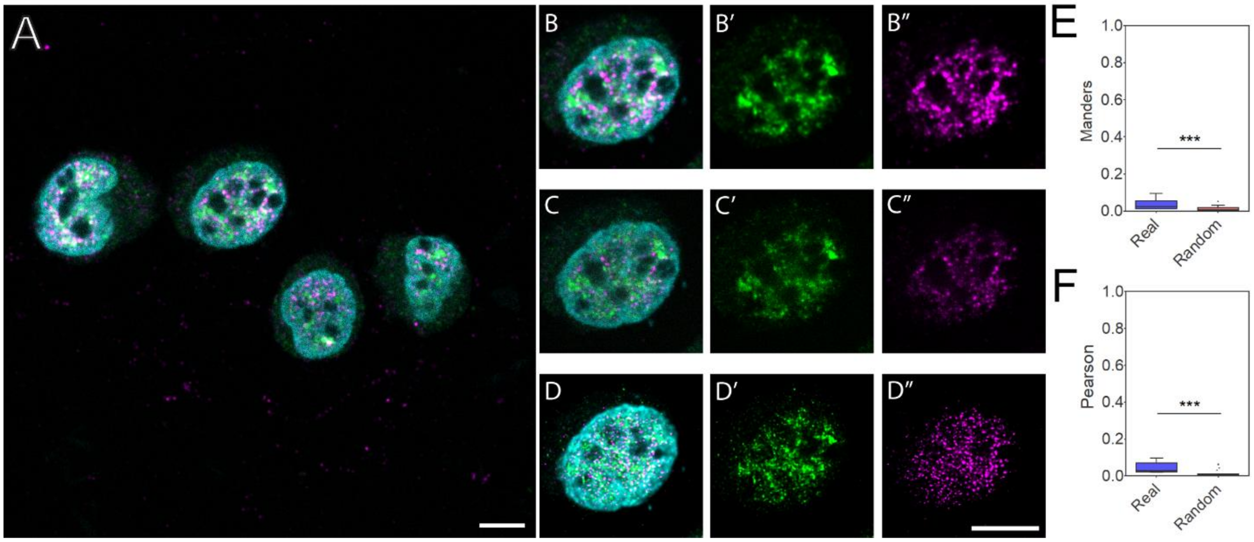

Supplementary figure 4. Imaging of SON and PI(4,5)P2 in FFPE U2OS-1 sections. Overview of the section with Hoechst nuclear stain in cyan, SON in green and PI(4,5)P2 in magenta (A), zoom in to a nucleus (B-D''), confocal (B-B'') and STED (C-C'') with deconvolution (D-D'') images merged (B, C, D), SON in green (B, B', C, C', D, D') and PI(4,5)P2 in magenta (B, B'', C, C'', D, D''). Manders overlap (E) and Pearson correlation (F) coefficients between PI(4,5)P2 and SON in real images and images in which first channel was 90° rotated with respect to the second channel (random). Scale bar 5 µm.
